# Feasibility of Using a Web-Based Nutrition Intervention Among Residents of Multiethnic Working-Class Neighborhoods

**Published:** 2007-06-15

**Authors:** Lorna H McNeill, Gary G Bennett, K Viswanath, Karen M Emmons, Elaine Puleo

**Affiliations:** University of Texas M.D. Anderson Cancer Center; Harvard School of Public Health/Dana-Farber Cancer Institute — Society, Human Development and Health; Harvard School of Public Health/Dana-Farber Cancer Institute — Society, Human Development and Health; Harvard School of Public Health/Dana-Farber Cancer Institute — Society, Human Development and Health; University of Massachusetts Amherst — Biostatistics

## Abstract

**Introduction:**

Using the Internet to promote behavior change is becoming more desirable as Internet use continues to increase among diverse audiences. Yet we know very little about whether this medium is useful or about different strategies to encourage Internet use by various populations. This pilot study tested the usefulness of a Web-based intervention designed to deliver nutrition-related information to and increase fruit and vegetable consumption among adults from working-class neighborhoods.

**Methods:**

Participants (N = 52) had access to the Web site for 6 weeks and received three e-mail reminders encouraging them to eat fruits and vegetables. The Web site provided information about overcoming barriers to healthy eating, accessing social support for healthy eating, setting goals for healthy eating, and maintaining a healthy diet, including recipes. We collected data on participants' use of the Web site, their Internet access and use, and their fruit and vegetable consumption.

**Results:**

The mean age of the participants was 46 years, 73% were white, 46% did not have a college degree, and 12% had household incomes at or below 185% of the federal poverty index. They reported consuming an average of 3.4 servings of fruits and vegetables per day. More than half of the participants owned a computer, 75% logged onto the Web site at least once, and those who visited the site averaged 3.8 visits and viewed an average of 24.5 pages. The number of log-ons per day declined over the study period; however, reminder e-mails appeared to motivate participants to return to the Web site. Roughly 74% of participants viewed information on goal setting, 72% viewed information on dietary tracking, and 56% searched for main course recipes.

**Conclusion:**

The results of this pilot study suggest that Internet-based health messages have the potential to reach a large percentage of adults from working-class neighborhoods who have access to the Internet.

## Introduction

Modern communication methods, such as the Internet and e-mail, have the potential to disseminate health information and engage large and varied audiences in health-promotion interventions (1,2). Even though access to the Internet varies by racial/ethnic group and socioeconomic level, the differences in Internet access are steadily narrowing. Recent studies suggest that an increasing percentage of racial/ethnic minority and working-class groups have Internet access (3,4). The Internet has the potential to deliver health information to diverse audiences both because of the ease, convenience, and immediacy with which it can convey information (5,6) and because it can reach audiences who prefer self-managed behavioral change interventions (7). As Internet access continues to increase among working-class and minority groups, knowledge about how to recruit these groups for Web-based behavioral-change programs, how to make such programs useful to them, and how to develop program features that promote their return visits will become increasingly important.

Research in social epidemiology has shown that living in poor, working-class environments is a risk factor for adverse health outcomes (8). Factors associated with living in working-class areas, such as lack of access to health-promoting services and increased exposure to crime and environmental hazards can explain some of the disparities in health outcomes. Working-class adults (defined as those who occupy nonmanagerial, low-paying, or low-status blue-collar occupations) have been shown to be less likely to engage in healthful behaviors (e.g., avoiding tobacco use, engaging in physical activity, eating a healthful diet) than those with higher job status and incomes (9-11) and to adopt health-promoting behaviors and reduce riskier behaviors at a slower rate (12). Communication may play a role in overcoming the barriers related to disparities in such social determinants of health (2), and Web-based interventions have shown promise in reaching working-class adults; in preventing and controlling conditions such as diabetes and obesity (13,14); and in promoting physical activity (15), healthful eating (16), and weight loss (7).

However, few studies have examined the effectiveness of Web-based interventions targeting working-class adults. Gustafson et al (17) studied the use of an interactive computer system that provided information on social-support and problem-solving techniques for women with breast cancer and found that lower-income women logged on more often and spent more time using the system than did more affluent women. Masucci et al (18) tested a telemedicine system among older, underserved patients at risk for cardiovascular disease and found that most participants used the system to report their health status. These studies, however, primarily involved participants with a disease or at high risk for a disease — people with a high motivation to participate in relevant interventions. They did not address the effectiveness of similar interventions among healthy populations, and such research that targets and recruits healthy people from working-class environments is needed in order to assess the effectiveness of Web-based interventions for this population (19).

In this pilot study, we assessed the effectiveness of a Web-based intervention in delivering nutrition-related information to adults residing in low-income, multiethnic neighborhoods in Boston, Massachusetts. Our primary aims were 1) to test the feasibility of enrolling adults from working-class neighborhoods in a Web-based intervention to increase fruit and vegetable consumption, 2) to examine strategies for encouraging people to visit and use the Web site, and 3) to assess participants' satisfaction with the Web site.

## Methods

The pilot study described here was conducted in the spring of 2004 as part of the Healthy Directions–Health Centers Study, a larger, randomized controlled trial of cancer prevention designed to reduce the prevalence of cancer risk factors among working-class adults seen at community health centers (20). The larger trial, concluded in 2003, was successful in increasing fruit and vegetable consumption among the participants; this pilot study was designed to assess the feasibility of using the Web — a lower cost alternative — to increase fruit and vegetable consumption among members of the target population. This study was conducted with institutional review board (IRB) approval from the Harvard School of Public Health and its affiliates.

### Participants

Participants in this study were drawn from a sample of 2219 adults who had participated in the larger intervention trial involving patients from 10 health centers in Boston. To be eligible for the larger intervention trial, patients had to live in a neighborhood in which, according to 2000 U.S. Census data, at least 66% of the population had working-class occupations, at least 20% of the population lived below the federal poverty level, or at least 25% of the population had not completed high school. Additional eligibility criteria for the study described here included being 18 to 60 years old; having access to the Internet at home, work, or any other venue; having an e-mail account or being willing to create an account; and being willing to participate in the study. We were interested in obtaining a sample of 50 adults to participate in the pilot study. Of the 2219 participants in the larger trial, 300 were randomly selected and invited to complete a short baseline phone survey; 123 (41%) of those selected completed the baseline survey; 89 (72%) of these 123 were deemed eligible to participate in the pilot study; and 52 (72%) of these 89 eligible people were then selected and enrolled.

### Study Procedures

After agreeing to participate, participants received a letter via postal mail introducing the study and informing them that a trained health counselor would contact them by phone to discuss their current dietary habits as indicated by their responses to the baseline survey. During this call, the health counselor encouraged participants to use the free study intervention Web site. Participants were provided access to the Web site for 6 weeks and received an introductory e-mail and three subsequent reminder e-mail messages sent at 2-week intervals encouraging them to use the Web site.

The introductory e-mail oriented participants to the study and provided them a hyperlink to the Web site. Participants were able to access the Web site by entering their e-mail address, which served as their unique identifier and was used to track their Web site activity. The introductory e-mail also informed participants that those who visited the Web site at least once would be entered into a raffle for a small incentive. The second e-mail was a general reminder, and the third provided tailored feedback on participants' fruit and vegetable consumption as indicated by their baseline survey responses and offered suggestions for how to increase their consumption. The final e-mail thanked participants for their time, named the two raffle winners, and reminded participants of the date that the Web site would be deactivated.

### Measures

Participants completed an interviewer-administered baseline telephone survey concerning their sociodemographic characteristics, their fruit and vegetable consumption during the previous 4 weeks, and their access to and use of computers and the Internet (including questions about their frequency of use, the location of the computer or computers they used, and their total time of use). To assess participants' fruit and vegetable consumption during the previous 4 weeks, we used the National Cancer Institute's 5 A Day for Better Health Program screener (21). In response to questions about the number of times per day that they ate fruits and vegetables, participants chose one of 10 precoded answers that ranged from "never" to "five times or more."  We then recoded their responses to obtain the average number of servings of fruits and the average number of servings of vegetables that they consumed per day and added these two numbers to obtain an average number of daily servings for fruits and vegetables combined.

The sociodemographic information collected included data on participants' race/ethnicity, age, sex, height and weight (which we used to calculate their body mass index [BMI; weight in kilograms divided by height in meters^2^]), education level, and yearly household income from all sources. We used this reported income data together with the number of household members supported by this income to determine whether participants' household income was at or below 185% of the U.S. poverty level as defined by federal poverty guidelines (22). Information about a participant's current or most recent job title was categorized into a three-category occupational status variable: working class (clerical, sales, skilled or unskilled labor), professional/managerial, or missing/unknown (category unable to be placed in either working class or occupational based on the title).

During the study, we collected objective data on participants' use of the intervention Web site by tracking the number of log-ins, the date and time of the log-ins, and the number of pages and hyperlinks selected by each participant.

### Description of the nutrition Web site

The Web site to which study participants were given access was based on the *5–9 a Day for Better Health *program, the National Cancer Institute's initiative to increase fruit and vegetable consumption among U.S. adults (www.5aday.gov). We modified the Web site for our target population by offering nontailored, yet culturally relevant and appropriate information on five major topics: overcoming barriers to healthy eating, getting social support for healthy eating, maintaining healthy eating habits, tracking one's eating habits, and setting reasonable and attainable nutrition goals. The Web site's home page included links to these topics and instructions on how to use the Web site, as well as links to more than 150 fruit and vegetable recipes, tips on how to increase one's fruit and vegetable consumption, and serving size information.

Participants were allowed to select any topic on the Web site in any order at any time. Each of the five topics began with a brief introduction, testimonials related to the topic, and additional links to explore that topic further. "Overcoming Barriers" provided examples of how to reduce barriers to healthy eating; "Family and Friends" encouraged participants to seek out members of their social network who could support their healthy eating attempts and provided tips on how to effectively reach out to them; "Setting Goals" described how to set specific nutrition goals; "Track What You Eat" described  the importance of using food diaries; and "Keep It Going" provided guidance on how to maintain healthy dietary practices.

### Data analysis

To carry out the analyses, we used SPSS (SPS Inc, Chicago, Ill) version 12.0 for Windows, and we used Chi-square testing to compare categorical variables for various demographic groups and the *t*-test to compare continuous variables; we considered differences to be statistically significant at a *P *value of 0.05 or less. We also report frequencies and descriptive statistics, including means and standard deviations.

## Results

### Participant characteristics

Participants' mean age was 46 years (standard deviation [SD] 9 years), and they had a mean reported annual household income of about $50,000 on which they supported an average of three people. Seventy-three percent were white, 73% were female, 46% did not have a college degree, and 12% had incomes at or below 185% of the federal poverty level. They consumed an average of 3.4 servings of fruits and vegetables per day; only 14% consumed at least 5 servings per day. Their mean BMI was 29.1 (Table); 71% were overweight or obese (i.e., had a BMI ≥25.0; data not shown).

### Internet and computer access

Of the 52 participants, 52% owned a home computer, 40% used a computer most often at home, 56% used one most often at work, and 35% used a dial-up telephone modem to access the Internet when at home. Participants reported spending an average of almost 2 hours per weekday on the Internet and roughly 3 hours per weekday on a computer. All participants used e-mail, as it was a requirement for participation in the study.

### Web site usage

Over the 6-week trial period, 39 (75%) of the 52 participants logged onto the Web site at least once, and those who did so visited the Web site an average of 3.8 times. Those who did not access the Web site did not differ significantly from those who did on any sociodemographic or behavioral characteristic. Of those who used the site, 74% viewed the site within the first 2 days of obtaining access to it, and 87% did so within the first week of gaining access. The number of log-ins to the Web site declined over the 6-week study period, although the reminder e-mails sent every other week did seem to motivate participants to return to the Web site. In the 2 weeks after the first e-mail reminder, 56% of those who used the site logged on; in the 2 weeks after the second reminder, 27% logged on; and in the single week after the final reminder, 56% logged on (Figure). 

**Figure 1:**
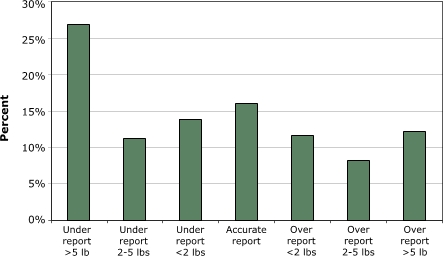
Percentage of 39 Web site users who accessed the site at various intervals.

**Table:** 

The Web site contained 192 distinct pages that were viewed 956 times in total. Participants who accessed the site viewed an average of 24.5 pages. Of the five main topics, "How to Set Goals" and "Tracking What You Eat" were viewed by the largest percentage of participants, 74% and 72%, respectively. The recipes pages were viewed by 56% of participants, and the section on "Family and Friends" was the least viewed Web page (39%). Although the number of pages viewed did not differ significantly by either baseline fruit and vegetable consumption or site of primary computer access (i.e., home, work, or other), on average, participants who reported that their primary Internet access was at work viewed slightly more pages than those who reported that their primary access was at home (28.0 versus 20.0 pages; *P* = .38), and those who reported consuming more than 3.5 servings of fruits and vegetables per day viewed almost twice the number of pages  as did those who consumed less than 3.5 servings (31.0 versus 19.0 pages; *P* = .20).

### Participants' evaluation of Web site

In the second e-mail, we asked participants to indicate which of the Web site features they liked the most and which they liked the least. Although only 20% of those who visited the site responded, most of those who did indicated that they liked the Web site, bookmarked it so that they could get quicker access to it, and printed out the pages they were most interested in so that they could refer back to them. Some suggested that the Web site could be improved through enhanced interactivity that would allow users to receive immediate feedback about their nutrition habits.

## Discussion

This 6-week intervention showed that adults living in working-class neighborhoods are interested in obtaining nutrition-related health information via the Internet and that many will use the Internet to access such information if they have the opportunity to do so. This finding indicates that the Internet can be an effective channel for conveying health information to this population.

Our first study aim was to test the feasibility of enrolling adults from multiethnic working-class neighborhoods in a Web-based intervention to promote fruit and vegetable consumption. We targeted and sought to enroll multiethnic working-class adults with low income and education levels who resided in such neighborhoods. In the larger intervention trial from which we recruited participants for this study, 40% of the participants were members of racial or ethnic minority groups (23,24), whereas only 14 (27%) of the 52 participants in our pilot study were. Similarly, participants in our pilot study were less likely to be poor than were members of the larger trial. Recruiting low-income participants to Web-based studies or surveys has been shown to be challenging, both because they are less likely to have access to the Internet and because they are more likely to prefer to use other communication sources (25). Previous studies of the effectiveness of Web-based programs in reaching diverse populations have shown mixed results (26), and we found no comparative  studies that assessed usage rates or user attitudes and preferences related to Web-based nutrition interventions, which indicates a need for continued research in this area. The fact that our recruitment efforts yielded a study population that was less poor and less ethnically and racially diverse than the cohort of the larger intervention trial indicates a need to find more effective strategies for recruiting multiethnic low-income people for studies of Web-based health interventions. Nevertheless, almost half of our study sample had less than a 4-year college degree, a group at higher risk for health problems than those with a degree. Moreover, Boston has one of the highest costs of living among U.S. cities, with few owner-occupied households and expensive monthly housing costs for renters (27). This high cost of living limits the amount of discretionary income that residents have for things such as computers; although potential participants in our study were required to have access to a computer in order to participate, only half owned a home computer. Our findings suggest, however, that the Internet is a promising avenue for reaching residents of largely working-class neighborhoods if they have Internet access.

Our second aim in this study was to examine strategies for encouraging people to visit the Web site. We found that 75% of study participants accessed the site, that almost 85% of those who visited the site first did so within the first week of the study, and that most participants who used the Web site viewed multiple topics. Participants seemed to be most interested in obtaining information on how to set goals for healthy eating and how to track their eating habits. Goal setting and self monitoring have both been shown to be effective strategies in diet and weight-control interventions (28,29), and our findings suggest that these interventions can be promoted effectively to residents of working-class neighborhoods through Web-based interventions. Participants were also interested in obtaining healthy recipes, particularly main courses that contained fruits or vegetables. We found that participants who consumed more than three servings of fruits and vegetables per day viewed slightly more Web pages than those who consumed less (although the difference was not statistically significant). This finding seems logical in that number of fruit or vegetable servings consumed per day and number of Web pages viewed are both indicative of interest in healthy eating. Other studies, however, have found that people who report poorer health and health-related behaviors are actually more likely to seek out health information (30).

Sending e-mails to prompt participants to continue their study participation has been used successfully in several studies (6,15,31). We similarly found proactive e-mails to be effective in prompting Web site use. In the 2 weeks after the initial and final e-mail reminders, more than half of the participants who had previously visited the Web site returned to it at least once; however, in the 2 weeks after the tailored e-mail, the third e-mail overall, only 26% of previous Web site visitors returned. The first e-mail reminder likely served its intended purpose of reminding participants to view the Web site, and the last reminded them of their final opportunity to do so. In general, these e-mails seemed to motivate previous users to return but were not effective in engaging those who never accessed the Web site.

### Strengths and limitations

Several limitations to this study should be noted. We did not attempt to reach those who did not access the site to ascertain why they did not. Thus we are unsure whether people changed their mind about participating after agreeing to do so, did not receive the welcome e-mail with the log-in information, or were otherwise unable to access the site. Similarly, we did not collect information on whether those who accessed the site received the e-mail reminders. There were suggestive findings (such as a relationship between where participants accessed the site and the number of pages they viewed) that were not statistically significant, probably because our study sample was too small to detect significant differences. Having a larger study sample should aid in determining whether such relationships are indeed significant. Future studies in which participants are provided access to an intervention Web site via e-mail should collect information about whether participants actually received the e-mail in order to assess intervention dose (15). Strengths of this study included our collecting objective data on participants' Web site usage rather than relying on participants to report the number and content of the Web pages they visited.

In this study, we allowed participants to access the Web site at their own time and pace and to tailor the intervention to their particular needs by selecting features of the Web site that were most relevant to them and ignoring those in which they had no interest. This latitude, though beneficial to study participants in that it allowed them to choose what material they wanted to receive and when and how often they wanted to receive it, could also be considered a study limitation in that it did not allow us to regulate the intervention dose. However, we were able to identify features of the Web site that seemed to engage participants, and these findings may have implications that will prove useful in future studies of Web-based interventions.

## Figures and Tables

**Table:** Selected Characteristics of Survey Population (N = 52), Boston, Mass, 2004

Characteristic	n (%)	Mean BMI (SD)	Mean servings/day of fruits or vegetables (SD)	Mean hours of self-reported Internet activity per weekday (SD)	Percentage of respondents who owned a home computer
**Age, y**					
21-39	15 (28.8)	26.7 (5.3)	3.6 (1.2)	2.1 (2.1)	60.0
40-49	15 (28.8)	28.5 (5.2)	2.7 (1.3)	1.4 (1.6)	60.0
50-60	22 (42.3)	30.7 (7.7)	3.5 (1.3)	2.1 (2.7)	40.9
**Sex**					
Male	14 (26.9)	30.3 (7.2)	3.4 (1.0)	2.4 (3.2)	50.0
Female	38 (73.1)	28.4 (6.4)	3.3 (1.4)	1.7 (1.8)	52.6
**Race/ethnicity**					
White	38 (73.1)	28.1 (6.6)	3.4 (1.3)	1.7 (2.3)	52.6
Black	8 (15.4)	28.9 (3.5)	2.9 (1.4)	2.5 (2.3)	50.0
Hispanic	5 (9.6)	32.3 (10.8)	3.4 (1.2)	2.2 (2.9)	40.4
American Indian	1 (1.9)	41.1	2.9	1.0	100.0
**Annual household income **					
$20,000-$29,999	4 (7.7)	30.9 (8.8)	3.0 (1.4)	2.5 (2.6)	50.0
$30,000-$39,999	5 (9.6)	27.5 (3.8)	3.6 (1.8)	1.1 (1.6)	40.0
$40,000-$49,999	8 (15.4)	23.9 (2.9)	3.0 (1.6)	1.6 (1.6)	50.0
≥$50,000	35 (67.3)	30.0 (6.8)	3.4 (1.2)	2.0 (2.3)	54.3
**Income in relation to federal poverty index **					
>185% of index	46 (88.5)	28.7 (6.5)	3.4 (1.3)	1.9 (2.3)	52.2
≤185% of index	6 (11.5)	30.4 (7.3)	2.6 (1.3)	1.7 (2.4)	50.0
**Education**					
High school or less	10 (19.2)	32.8 (6.1)	2.9 (1.5)	1.3 (1.5)	30.0
Less than 4 years of college	14 (26.9)	27.3 (5.5)	3.0 (0.8)	2.4 (3.5)	64.3
4 years of college	8 (15.4)	26.1 (6.7)	3.0 (1.4)	1.7 (1.7)	62.5
Graduate school	18 (34.6)	30.2 (6.7)	3.8 (1.2)	2.0 (2.0)	44.4
Missing/unknown	2 (3.8)	20.9 (1.4)	4.7 (1.6)	2.0 (0.0)	100.0
**Occupational status**					
Professional/managerial	34 (65.4)	9.1 (5.9)	3.6 (1.3)	1.8 (2.4)	50.0
Working-class	15 (28.8)	27.2 (5.4)	2.9 (1.3)	2.2 (2.0)	60.0
Missing/unknown	3 (5.8)	34.9 (15.2)	2.3 (0.5)	0.6 (0.5)	33.3
**Overall**	**52 (100.0)**	**29.1 (6.6)**	**3.4 (1.3)**	**1.9 (2.3)**	**51.9**

BMI indicates body mass index (weight in kilograms divided by height in meters squared).
